# Open reduction and internal fixation of extracapsular mandibular condyle fractures: a long-term clinical and radiological follow-up of 25 patients

**DOI:** 10.1186/1471-2482-14-68

**Published:** 2014-09-07

**Authors:** Alessia Spinzia, Renato Patrone, Evaristo Belli, Giovanni Dell’Aversana Orabona, Claudio Ungari, Fabio Filiaci, Alessandro Agrillo, Giacomo De Riu, Silvio Mario Meloni, Gianmauro Liberatore, Pasquale Piombino

**Affiliations:** 1 Maxillo-facial Surgery Unit, IRCCS University Hospital San Martino-IST National Institute for Cancer Research, Genova, Italy; 2 Maxillofacial Surgery Department, Federico II University of Naples, Via Pansini, 5, 80131 Naples, Italy; 3 Maxillofacial Surgery Department, Sant’Andrea Hospital, “Sapienza” University of Rome, Rome, Italy; 4 Maxillofacial Surgery Department, Policlinico Umberto I of Rome, “Sapienza” University of Rome, Rome, Italy; 5 Maxillofacial Surgery Department and Dentistry Department, University Hospital of Sassari, Sassari, Italy; 6 Maxillofacial Surgery Department, Azienda Ospedaliera Universitaria Pisana of Pisa, Pisa, Italy

**Keywords:** Extracapsular mandibular condyle fractures, Open reduction and rigid internal fixation, Clinical and radiological follow-up, Mandibular fracture

## Abstract

**Background:**

During the last 2 decades, many studies on the treatment of mandibular condyle fracture have been published. The incidence of mandibular condyle fractures is variable, ranging from 17.5% to 52% of all mandibular fractures. This retrospective study evaluated the long-term clinical and radiological outcomes after surgical treatment of 25 patients with a total of 26 extracapsular condyle fractures.

**Methods:**

We used 2 types of surgical approaches, the retromandibular retroparotid or preauricular approach. Three kinds of rigid internal fixation plates were used—single plate, double plate, and trapezoidal plate. The following post-operative clinical parameters were evaluated: dental occlusion, facial nerve functionality, skin scarring, and temporomandibular joint functionality. All patients underwent post-operative orthopanoramic radiography and computed tomography. The patients were also monitored for complications such as Frey’s syndrome, infection, salivary fistula, plate fracture, and permanent paralysis of the facial nerve; the patient’s satisfaction was also recorded.

**Results:**

Of the 25 patients, 80% showed occlusion recovery, 88% had no facial nerve injury, and 88% presented good surgical skin scarring. The patients showed early complete recovery of temporomandibular joint functionality and 72% of them were found to be asymptomatic. The postoperative radiographs of all patients indicated good recovery of the anatomical condylar region, and 80% of them had no postoperative complications. The average degree of patient satisfaction was 8.32 out of 10. Our results confirm that the technique of open reduction and internal fixation in association with postoperative functional rehabilitation therapy should be considered for treating patients with extracapsular condylar fractures.

**Conclusion:**

The topic of condylar injury has generated more discussion and controversy than any other topic in the field of maxillofacial trauma. We confirm that open reduction and internal fixation is the treatment of choice for patients with neck and sub-condylar mandibular fractures.

## Background

During the last 2 decades, many studies on the treatment of mandibular condyle fracture have been published. However, the timing and methodology of treatment are still widely debated, despite the advent of new technologies, such as advanced computed tomography (CT), and new materials such as titanium fixation devices.

The incidence of mandibular condyle fracture is variable, ranging from 17.5 to 52% of all mandibular fractures [1]. The main causes of this type of fracture are road traffic accidents (approximately 50%), falls (30%), and personal violence (20%) [2]. In addition, age, gender, and the cause of fractures show a statistically significant association with the incidence of mandibular condyle fractures, with bicycle accidents (24.61%), car accidents (23.07%), and falls (23.07%) being the most common causes of such fractures among women [1,2].

Mandibular condyle fractures are categorized into 2 groups: intra- or extra-capsular fracture; this categorization is based on the anatomical aspects such as the condylar head, condylar neck, and subcondylar region. Another classification method is based on the condyle position, i.e., undisplaced, deviated, displaced (with medial or lateral overlap or complete separation), or dislocated (outside the glenoid fossa) condyle fractures [1].

Further, sagittal or diacapitular mandibular condylar fractures are very rare and difficult to identify via conventional radiography. These fractures do not require any surgical treatment but require early mobilization [3,4]. Therefore, these cases were excluded, as they do not fall under the scope of this study.

A variety of treatment options are available according to the clinical symptoms and diagnostic findings of the fracture, e.g., unilateral or bilateral fracture, displacement, dislocation, size and position of the condylar segment, dental malocclusion, mandibular dysfunction, and patient’s willingness to receive surgical treatment. Other important parameters that may affect the final treatment choice are the surgeon’s experience, patient’s age, and general health status [1].

Skin surgical approaches as well as fixation methods are still highly debated although the number of proponents of surgical treatment has been gradually increasing throughout the last decade. The majority of the papers published in a recent 5-year period (2006–2011) have discussed and recommended the surgical treatment of condylar fractures rather than using conservative approaches [1,3,5].

Several studies have focused on the absolute and relative indications for the open reduction of mandibular condylar fractures. Zide and Kent described what was considered the “gold standard” treatment during the early 1980s (see the Zide and Kent’s indications for open reduction section) [6]. Obviously, the indications for surgery versus conservative treatment were based on the materials and surgical techniques available at that time.

### Zide and Kent’s indications for open reduction (1983)

1. Absolute

• Displacement into middle cranial fossa

• Impossibility of obtaining adequate occlusion by closed reduction

• Lateral extracapsular displacement

• Invasion by foreign body

2. Relative

• Bilateral condylar fractures in an edentulous patient without a splint

• Unilateral or bilateral condylar fractures where splinting cannot be accomplished for medical reasons or because physiotherapy is impossible

• Bilateral condylar fractures with comminuted midfacial fractures, prognathia or retrognathia

• Periodontal problems

• Loss of teeth

• Unilateral condylar fracture with unstable base

With the application of rigid internal fixation (RIF) techniques to the cranio-maxillofacial skeleton in the mid-1980s, new indications and contraindications have slowly evolved on the basis of perceived advantages or disadvantages of one technique over another. This transition can be observed through the numerous attempts by various authors to formulate clear indications for the surgical treatment of mandibular condylar fractures [6-9]. Several approaches have been proposed: the preauricular approach followed by retroauricular, submandibular, coronal, or intraoral incision or a combination of these approaches [1].

In the last few years, some authors have considered another method: transoral endoscopic-assisted open reduction. This method is a valid alternative to the transcutaneous approach for the reduction and fixation of extracapsular condyle fractures in selected cases [10]. With regard to fracture fixation, the use of numerous devices and methods has been reported, ranging from external fixation to rigid internal fixation. Only a few authors have reported the long-term clinical and radiological follow-up details exclusively after surgical treatment of mandibular condylar fractures [11-15]. After performing routine surgical treatment of mandibular condylar fractures for several years, we reviewed our case series and performed a retrospective study to present our long-term clinical and radiological findings.

## Methods

In the period between 2003 and 2011, 40 patients visited our clinic for the surgical treatment of mandibular condyle fractures. The inclusion criteria for this study were as follows: adolescent or adult patients in good health, presence of neck and subcondylar mandibular fractures associated with post-traumatic dental malocclusion, and alteration of the temporomandibular joint functionality on radiological examination. Edentulous patients and patients below the age of 15 years were excluded. All the 40 patients were contacted by telephone and invited to volunteer for clinical and radiological examinations, but only 25 patients accepted our invitation and were finally included in this study. The study involved 18 males (72%) and 7 females (28%) (male/female ratio, 2.5/1), and the age at the time of injury ranged from 16 to 55 years (mean age, 27 years). The most frequent causes of injury were road traffic accidents (60%) followed by accidental falls (32%) and personal violence (8%). The mean follow-up duration was 3.67 years (range, 1–10 years).

The 25 patients reported a total of 28 mandibular condylar fractures: 22 (88%) unilateral condylar fractures and 3 (12%) bilateral condylar fractures of which 26 were surgically treated. The patients were evaluated by the authors according to a detailed protocol. The preoperative radiographic examinations comprised panoramic radiography and CT (high-definition 1-mm thickness, high-resolution 3D reconstruction) to determine the degree of condylar displacement. We used 2 different types of surgical approaches, retromandibular retroparotid [16] or preauricular approach [17], depending on the severity of the fracture.

According to Lindahl’s classification [18] of mandibular condylar fractures, we used the preauricular approach [17] in cases of condylar neck fractures and the retromandibular retroparotid approach [16] in cases of subcondylar fractures.

The retromandibular retroparotid approach [16] was performed through a 3- to 4-cm vertical incision that extended inferiorly from the tip of the mastoid, below the ear lobe, anterior to the sternocleidomastoid muscle. The incision was made in the anteromedial direction, beneath the parotid gland, toward the posterior border of the mandible until the incision reached the condylar fracture. The branches of the facial nerve were not encountered. The preauricular approach [17] was performed through a 5- to 6-cm skin incision extending superiorly to the top of the helix leading to an anterior temporal extension. Then, the temporal fascia (superficial layer) was incised in the vertical direction, and a blunt dissection was performed, exposing the lateral part of the temporomandibular joint capsule.

By remaining beneath the superficial layer of the temporal fascia, the temporal branches of the facial nerve were avoided. The temporomandibular joint capsule was incised, and the condylar fracture was exposed. Three different types of rigid internal fixation plates were used: the single plate, double plate, and trapezoidal plate. Eight fractures (30.7%) were treated using 4-hole 2.0-mm single plates (SYNTHES Maxillofacial, Paoli, PA, US), 10 fractures (38.6%), using 4-hole 2.0-mm double plates (SYNTHES) and the remaining 8 fractures (30.7%), using 4-hole-Modus TCP**®** (Trapezoidal Condylar Plates, Medartis, Basel, Switzerland). Of the 25 patients, 11 practiced post-surgical functional therapy: 8(32%) received physiotherapy and 3 (12%) received therapy with a modified Balters’ bionator.

The patients were trained to perform a set of exercises consisting of forced active and passive mouth opening. The training was aimed to correct the jaw’s alignment to achieve a satisfactory range of movements. The patients treated for unilateral condylar fracture were instructed to stand in front of a mirror and apply gentle force using their fingers to open the mandible along a straight line, using the upper interincisive line as the reference line; in addition, the gradual recovery of the normal range of jaw movement was encouraged. Use of left and right laterality, with particular attention to the movement of the side contralateral to the fractured side, was recommended. Furthermore, the rehabilitation of protrusion as well as correction of lateral mandibular deviation of the fractured side was encouraged. The patients treated for bilateral condylar fracture received post-surgery physiotherapy in the same manner described above. We suggest particular attention be paid during protrusive mandibular movement. The patients were instructed to perform the exercises 3 times a day, with 10 minutes spent for each movement. In cases where the Balter’s bionator was used, the device was built by taking the construction bite in maximum protrusive in case of bicondylar fracture and in contralateral laterality in the case of unilateral condylar fracture. The patients were recommended to use it for as long as possible every day.

The following post-operative clinical parameters were monitored: (1) dental occlusion, (2) facial nerve functionality according to the House-Brackmann Facial Nerve Grading System [19], (3) skin scarring according to the Vancouver Scar Scale (VSS) of Baryza [20], (4) postoperative temporomandibular joint functionality, and (5) postoperative symptomatology according to the Research Diagnostic Criteria for Temporomandibular Joint Disorders (RDC/TMD) [21]. Finally, the patient’s satisfaction concerning the treatment received was evaluated. The degree of satisfaction was quantified by asking the patient to rate the treatment received using a score from 0 to 10.

Habitual occlusion recovery was assessed by asking the patient whether he/she perceived his/her occlusion to be the same as that experienced before the trauma.

The postoperative functionality of the facial nerve was evaluated using the House-Brackmann Facial Nerve Grading System [19]. The House-Brackmann system is used to score the degree of damage in facial nerve palsy. The score is determined by measuring the upwards (superior) movement of the mid-portion of the top of the eyebrow and the outward (lateral) movement of the angle of the mouth. Each reference point corresponds to 1 point for each 0.25-cm movement, up to a maximum of 1 cm. The scores are then added to obtain the maximum score out of 8 [19].

The VSS [20] is the most widely used scar assessment instrument. This scale, originally developed to rate burn scars, is a standardized grading instrument based on the following 4 parameters: pliability, pigmentation, vascularity, and scar height, all of which are evaluated independently. The total score (range 0 to 13) was obtained by adding the scores for each of the 4 parameters. The lower the score the greater the resemblance of the scar tissue to normal tissue.

The RDC/TMD [21] consists of 2 axes: axis I (clinical examination, evaluation, and diagnosis) and axis II (behavioral questionnaires). Axis I was carefully compiled after scrupulous measurements taken using a caliper of the maximum opening without pain, maximum opening, passive opening, overbite, right lateral movement, left lateral movement, protrusion, deviation from the midline, and opening pattern.

We completed a historical review by asking the patients whether they experienced any facial pain homolateral to the side of the fracture and the exact position of this pain. The presence of temporomandibular joint pain, facial muscular pain, and combination of such pain was taken into account. Axis II was completed by the patients but was not taken into account in this study.

The presence of postoperative complication such as Frey’s syndrome [22], infection, salivary fistula, plate fracture, and permanent paralysis of the facial nerve were taken into account.

After surgery, the mandible, the correct anatomical restoration of the fractured site, and the possible presence of plate fracture or screw loosening were evaluated via orthopanoramic radiography and high-definition CT. The patients photographs before and after surgery were also evaluated.

All patients granted written specific consent for all photographs and personal data to be used in every medical pubblications, journal, textbook and electronic pubblications. The investigation was conducted according to the ethical principles of the 1975 Helsinki Declaration for biomedical research involving human subjects, as revised in 2004. The present work represents a retrospective study, so that it did not require ethical comitte approval; its design, inclusion and exclusion criteria, and treatment protocol were reviewed and approved by the Research Committee of senior attending surgeons of the department.

## Results

Of the 25 patients, 5 (20%) reported postoperative occlusal disturbance due to premature dental contact whereas the remaining 20 (80%) had normal occlusion. Six months after surgery, 10 (40%) patients reported slight weakness of the facial nerve upon careful inspection (grade II on the facial nerve grading system). However, after 3 years, none of the patients showed any signs of facial nerve involvement (Table 1).

**Table 1:** Facial nerve score at 3 years post-operative

**Facial nerve score at 3 years post-operative**					
**Grade I**	**Grade II**	**Grade III**	**Grade IV**	**Grade V**	**Grade VI**
25	0	0	0	0	0

According to the VSS [20], of the 25 patients who had undergone surgery, 5 (19%) had a total score of 0, corresponding to the score for individuals with normal tissue. In addition, 23 (88%) had a total score of ≤6, 3 had a total score of 5, and 1 had a total score of 6. Only 2 patients had a total score of 7, and the high value of 9 was recorded in only 1 case. The highest possible score (13) was not reported by any patient (Table 2).

**Table 2:** Total Vancouver Scar Scale score assessment of the surgical scar

**Vancouver scar scale total scores of surgical scare**														
**Total score**	**0**	**1**	**2**	**3**	**4**	**5**	**6**	**7**	**8**	**9**	**10**	**11**	**12**	**13**
**Number of surgical approach**	5	4	2	3	5	3	1	2	0	1	0	0	0	0

In the patients treated for unilateral condylar extracapsular fracture, the lateral movement of the side contralateral to the fractured side was between 2 mm and 13 mm (average, 7.1 mm), the lateral movement of the fractured side was between 4 mm and 14 mm (average, 9.3 mm), and the protrusion was between 2 mm and 9 mm (average, 4.5 mm).

In the patients treated for bilateral condylar extracapsular fracture, the right lateral movement was between 6 mm and 12 mm (average, 9 mm), the left lateral movement, between 6 mm and 9.5 mm (average, 8.1 mm), and the protrusion, between 2 mm and 7 mm (average, 4.3 mm).

In all the 25 patients, the maximum opening without pain was between 18 and 50 mm (average, 35.48 mm), the maximum opening, between 19 mm and 54 mm (average, 40.8 mm), and the passive opening, between 21 mm and 60 mm (average, 42.28 mm). The values reported in accordance with the physiological function values of mandibular movement recommended by Okeson [23] are presented in Table 3.

**Table 3:** Post-operative mandibular motion values, according to Okeson physiological values 23

**Post-operative mandibular motion values in the unylateral condylar fractures**	
**Physiological values of the mandibular motion**	**Number of patients**
**Maximal mouth opening** (mm)	
> 40 mm	15 patients (62,5%)
< 40 mm	9 patients (37,5%)
**Lateral movement controlateral to the side of the fracture** (mm)	
> 8 mm	13 patients (54%)
< 8 mm	11 patients (46%)
**Lateral movement homolateral to the side of the fracture** (mm)	
> 8 mm	12 patients (50%)
< 8 mm	12 patients (50%)
**Maximal protrusion** (mm)	
> 8 mm	2 patients (8%)
< 8 mm	22 patients (92%)

In the case of the patient treated for bilateral extracapsular condylar fracture, we reported the following values: maximal mouth opening, 42 mm; left lateral movement, 9.5 mm; right lateral movement, 9 mm; and protrusion, 4 mm. Concerning the mouth opening pattern (Table 4), of the 15 patients treated for right condylar fracture, 5 (33.3%) had the opening pattern type 0, 2 (13.3%) had type 1, 2 (13.3%) had type 2, 5 (33.3%) had type 3, and 1 (6.6%) had type 4. Of the 9 patients treated for left condylar fracture, 2 (22%) had an opening pattern type 1, 1 (11%) had type 2, 5 (55%) had type 3, and 1 (11%) had type 4. The patient treated for bilateral extracapsular condylar fracture had a mouth opening pattern type 0. With regard to facial pain, 18 (72%) of the 25 patients treated were asymptomatic (Table 5). The following postoperative complications were reported: plate fracture, 0; permanent paralysis of the facial nerve, 0; Frey’s syndrome, 3 (two instances reported in the same patient) [22]; salivary fistula, 0; and infection, 1 patient (Table 6).

**Table 4:** Mouth opening pattern

**Mouth opening pattern**	
**Description of pattern**	**Type of pattern**
Straigh	0
Right lateral deviation (uncorrected)	1
Right corrected	2
Left lateral deviation (uncorrected)	3
Left corrected	4
Other	5

**Table 5:** Facial pain grade: 0 = asymptomatic; 1 = temporomandibular joint pain homolateral to the side of fracture; 2 = muscolar facial pain homolateral to the side of fracture; 3 = both

**Facial pain**	
**Grade**	**Number of patients**
**0**	18 (72%)
**1**	3 (12%)
**2**	0 (0%)
**3**	4 (16%)

**Table 6:** Post-operative complications after surgical treatment of condylar fractures

**Post-operative complications**	
**Type of complications**	**Number of complication**
**Frey’s syndrome **^**22**^	3
**Fracture of the plate**	0
**Infection**	1
**Salivary fistula**	0
**Permanent paralysis of the facial nerve**	0

In all cases, the postoperative radiographic follow-up of the 25 patients (100%) indicated a good restoration of the condylar mandibular anatomy, the correct positioning of the mandibular condyle in the glenoid fossa, and the absence of screw loosening and plate fracture.

The satisfaction degree of the patients concerning the received treatment was between 4 and 10 (average value: 8.32).

## Discussion

Condylar injury is the most controversial subject in the field of maxillofacial trauma. Previously, conservative management of condylar fractures was favored. Troulis and Eckelt et al. reported that closed treatment remains the favored approach in several centers [24,25]. Ellis et al. emphasized that the potential risks of open reduction and internal rigid fixation (ORIF) must be weighed carefully against its potential benefits [26].

Although the correct therapy for mandibular condylar fractures in adult patients is still a topic of debate, many surgeons now favor open treatment of displaced condylar fractures as the method involving reduction and rigid fixation allows for good anatomic repositioning. Surgical therapy is generally adopted in cases where a conservative treatment would not ensure a suitable “restitutio ad integrum” of the morpho-functional site of the fracture.

Numerous attempts have been made over the years to identify the indications for the surgical treatment of condylar fractures, with the relative and absolute indications of Zide and Kent [6] being the most widely cited and used. However, none of these indications has been fully accepted until now, and the choice of treatment is currently still widely debated.

The important factors that should be considered before opting for surgical treatment of mandibular condylar fractures are the patient’s age, the general clinical conditions, the level and degree of the fracture fragment displaced/dislocated, degree of dental occlusion, and temporomandibular joint functionality.

The patient’s age and general clinical condition are the main elements that should be considered. A CT scan (axial, coronal, and 3D reconstruction) highlights the exact level of the fracture and degree of condylar displacement. Careful intraoral clinical examination is required for examining the degree of dental occlusion and temporomandibular joint functionality. We suggest that mandibular condylar fracture requires surgical intervention when 5 key elements “co-exist” as shown in the section of our indications for open reduction and rigid internal fixation.

### Our indications for open reduction and rigid internal fixation

Adolescent or adult patient

Optimal general clinical conditions

Radiological signs of presence of the neck and sub-condylar fracture

Dental malocclusion

Alteration of the temporomandibular joint functionality

Following Lindahl’s classification, we used 2 types of surgical approaches: the preauricular approach [17] in cases of neck condylar fractures and the retromandibular retroparotid approach [16] in cases of subcondylar mandibular fractures [18].

With regard to the fixation, in addition to the use of a single plate, we have always preferred using the double plate or trapezoidal plate in accordance with the literature findings, as they provide greater stability than other types of osteosynthetic implants [13,27-29].

We treated 8 fractures (30.7%) using 4-hole 2.0-mm plates, 10 fractures (38.6%) using 4-hole 2.0-mm double plates and the remaining 8 fracture (30.7%) using 4-hole Modus TCP**®**. These osteosynthetic implants have produced adequate long-term results as demonstrated by the absence of complications such as plate fracture or screw loosening at the follow-up examination.

**Figure 1:**
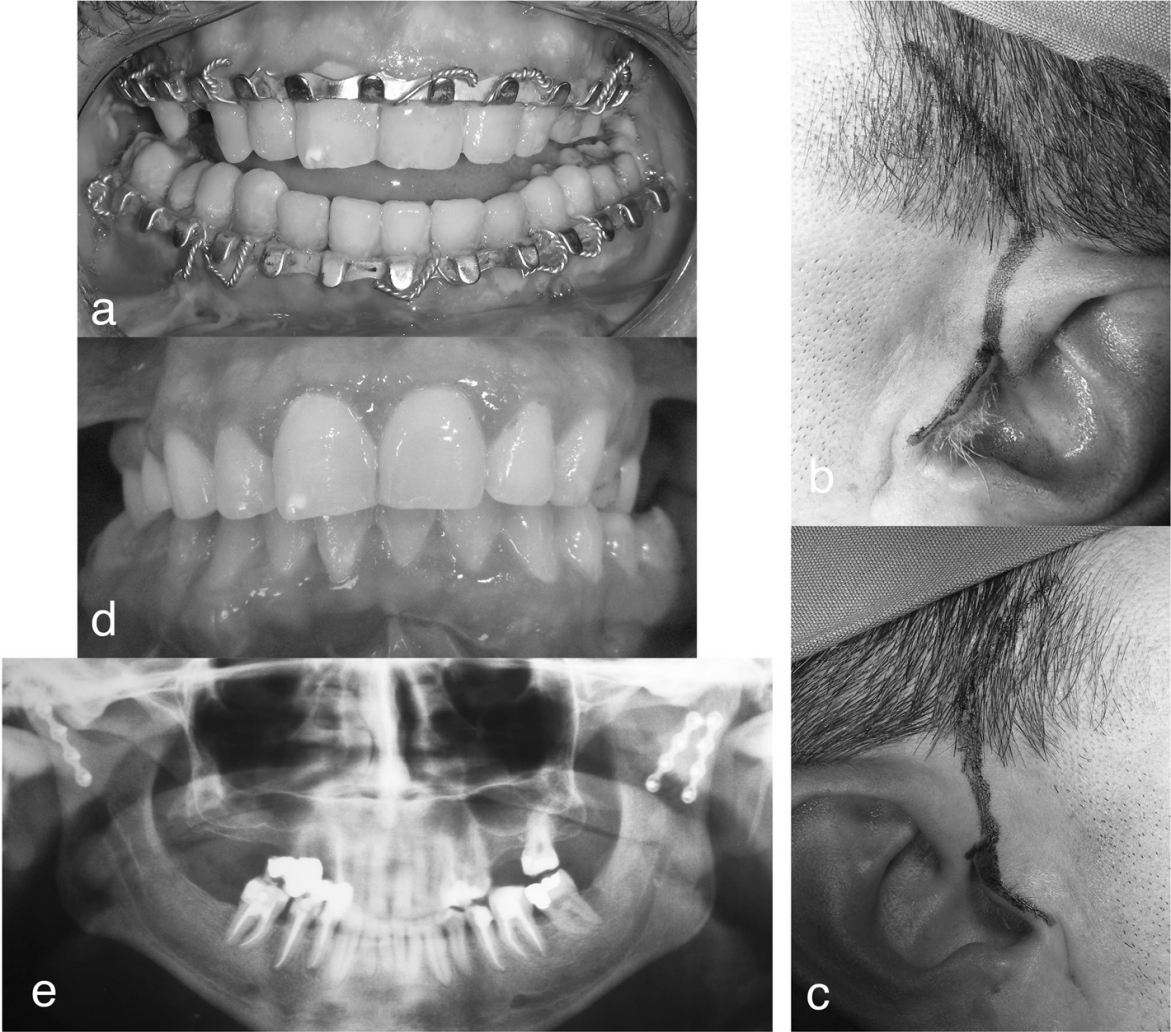
Mandibular sub-condylar bilateral fracture: a) anterior open-bite with a posterior precontact; b) left preauricolar approach; c) right preauricolar approach; d) post-surgey occlusal plane; e)post-surgery orthopantomography.

Twenty-five patients were examined with an average follow-up period of 39 months (approximately 3 years). As specified in the literature [9,10,12,30], a satisfying occlusion was observed in all patients (Figure 1). Grinding of the teeth was performed by the 5 patients with postoperative occlusal disturbance caused by premature contact.

Jensen et al. [9] reported that minor adjustment of the postoperative occlusion was necessary in 6 of 15 patients. Gonzalez-Garcia et al. [10] observed minimal occlusal changes in 1 of the 17 patients that underwent endoscopy-assisted surgery. Iizuka et al. [12] reported a centric occlusion in all 27 patients treated surgically without fixation. Leiser et al. [30] observed satisfactory occlusion in 10 patients that were surgically treated.

In patients with unilateral condylar fractures, the average lateral movement of contralateral side of the fracture was 7.1 mm, and the average homolateral movement of the fracture side was 9.3 mm, with no substantial differences between the 2 sides. These data indicate good restoration of the lateral movements of the mandible, with a slight deficit in the lateral movement of the contralateral side (in accordance with the clinical picture of these fractures).

In patients with bilateral condylar fractures, the average value of the right lateral movement was 9 mm and the average value of the left lateral movement was 9.5 mm. These data indicate good restoration of the lateral movements of the mandible.

The average value of protrusion in patients with a unilateral fracture was 4.5 mm, while in patients with bilateral condylar fracture, it was 4.3 mm. In all 25 patients, the average value of the maximum opening was 40.8 mm.

**Figure 2:**
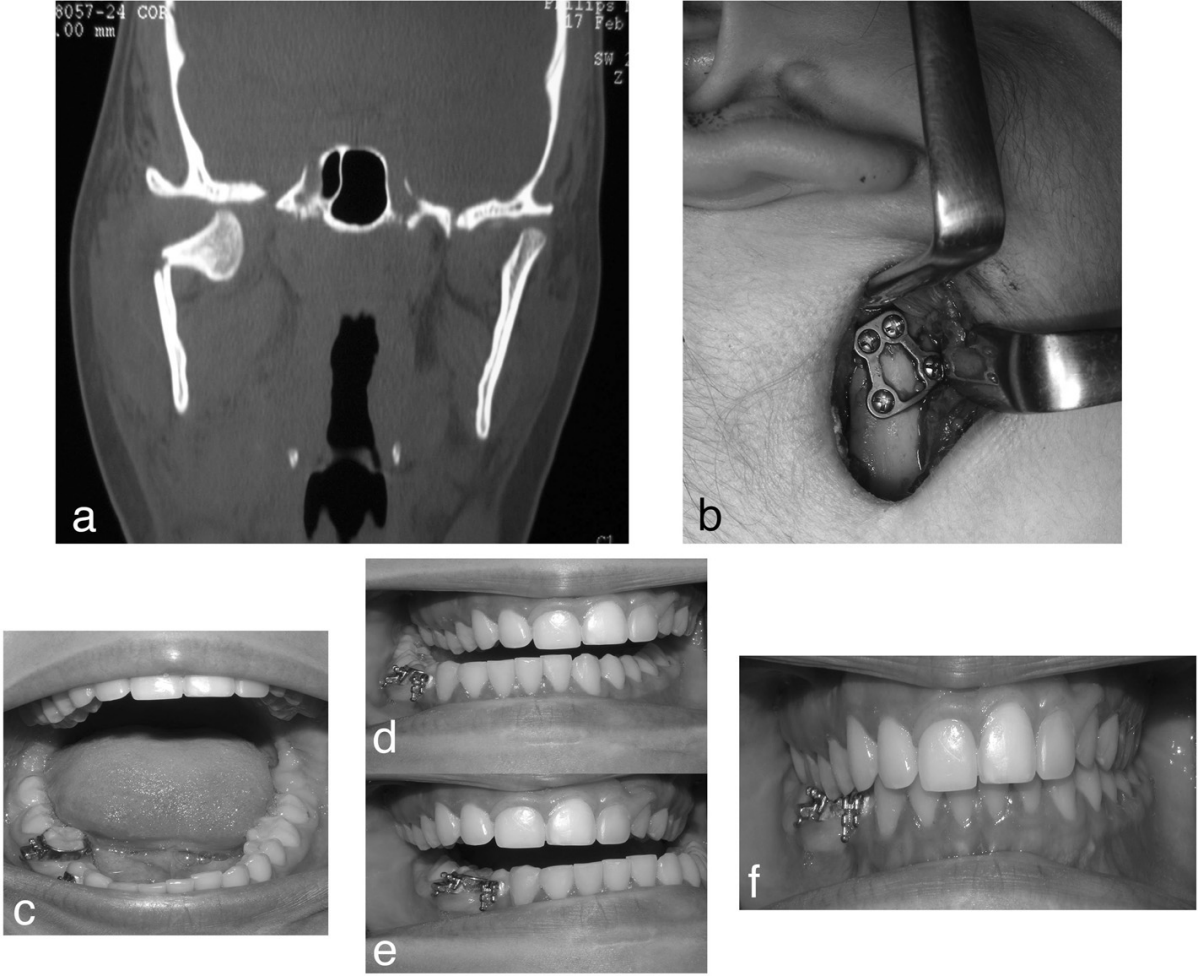
Right mandibular sub-condylar fracture: a) pre-surgery CT (coronal view); b) ORIF with a TCP plate; c) mouth opening; d) right lateral movements; e) left lateral movements; f) post-surgey occlusal plane.

According to the physiological values of mandibular motion recommended by Okeson [23], we found that our patients presented early complete restoration of all mandibular movements after surgery, and only the protrusion was limited in 2 patients who were surgically treated (Figure 2). Corresponding to our findings, Gonzalez-Garcia et al. [10] and Iizuka et al. [12] reported early complete restoration of mandibular movements in patients treated for mandibular condylar fractures.

With regard to mouth opening patterns, a small percentage (24%) of surgically treated patients showed a straight pattern. We found that 72% of the patients were asymptomatic for facial pain. Thus, we can confirm that facial pain is a not common consequence of ORIF.

**Figure 3:**
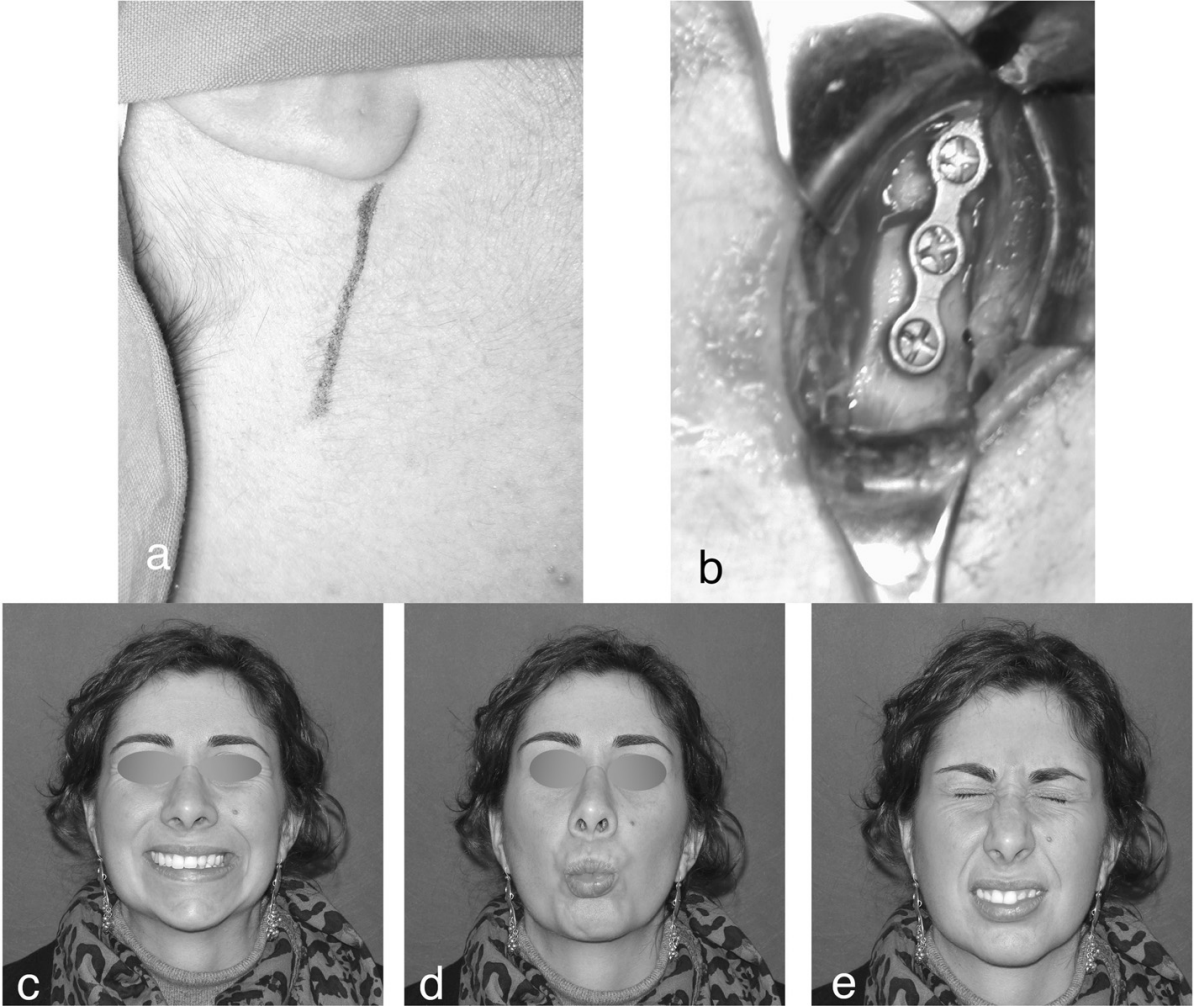
Right mandibular sub-condylar fracture: a) retromandibular approach; b) ORIF with a single plate; c,d,e) facial nerve functionality.

In general, the most feared risk of surgical treatment for condylar fractures is facial nerve injury. The likelihood of facial nerve injury was evaluated in our series according to the House-Brackmann Facial Nerve Grading System [19]. This grading scale is considered to accurately describe a patient’s facial function and monitor patient status over time for assessing the course of recovery. It was developed as a rough scale, with the objective of placing patients in general categories. Therefore, it has wide applications and is reliable. In this study, temporary facial nerve weakness (grade II per House-Brackmann system) occurred in 10 patients, observed 6 months after surgery (Figure 3). This complication may be the result of intraoperative soft tissue stretching probably caused by the rapid recovery of facial nerve functionality. We observed that temporary facial nerve weakness occurred more frequently in fractures that were treated using the retromandibular retroparotid approach [16], which requires extensive stretching of the marginalis mandibulae nerve when exposing the condylar region. In fact, 7 of the 10 patients with temporary facial nerve palsy were treated using the retromandibular retroparotid approach [16] and 3 patients, using the preauricular approach [17]. However, none of the patients showed permanent damage to the facial nerve. Our results show a low incidence of facial nerve injury associated with this approach, meaning it is the preferable approach when there is an indication for ORIF in mandibular condylar fractures. No damage to the facial nerve was observed [9,10,12,30], indicating that it is a safe and reproducible procedure.

**Figure 4:**
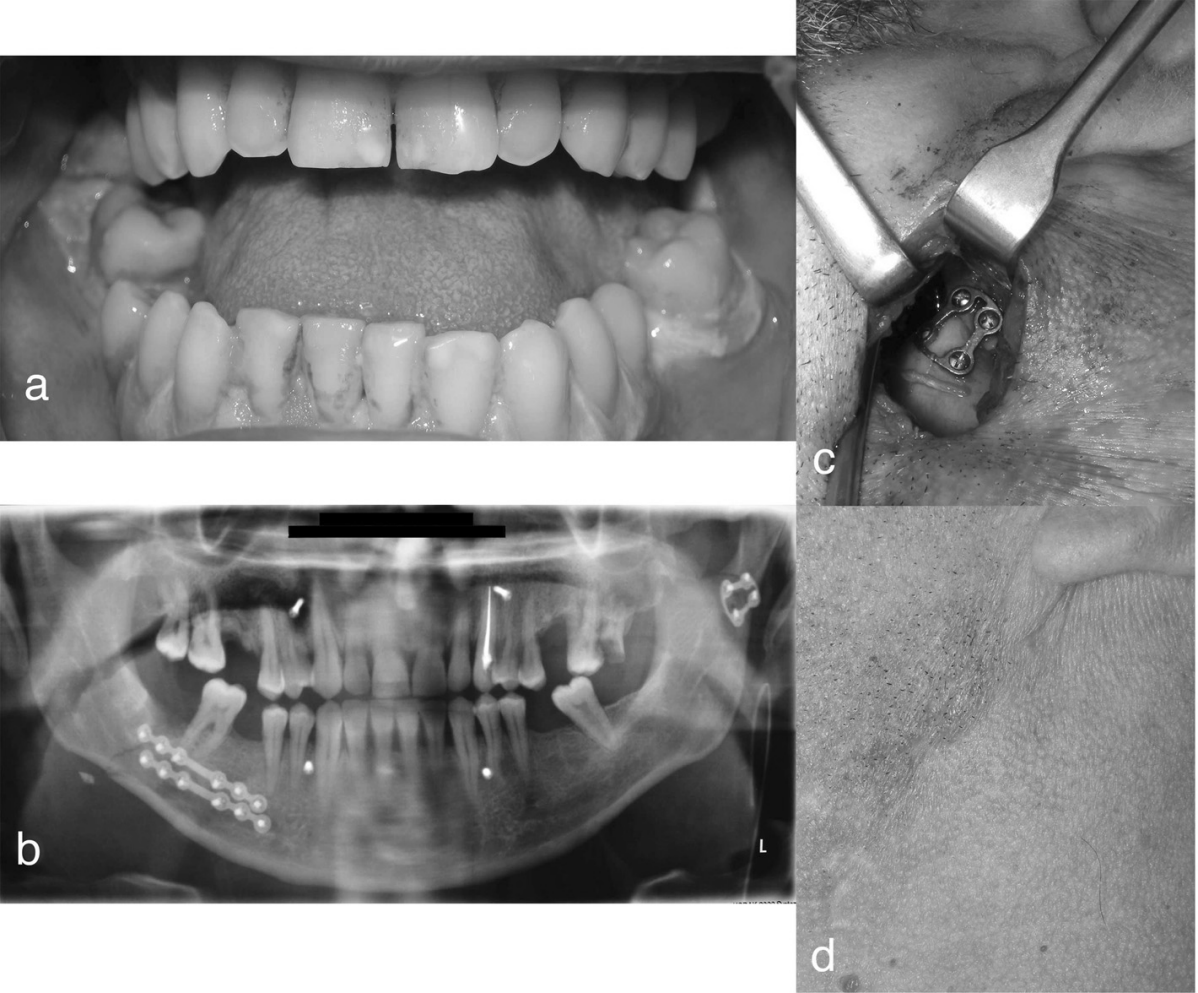
Left mandibular sub-condylar fracture: a) pretrauma mouth opening; b) post-surgery orthopantomography; c) ORIF with a TCP plate; d) scare result.

According to the literature [9,10,12,30] and the VSS scores [20], we observed particularly positive outcomes when the preauricular [17] and retromandibular retroparotid [16,31] approaches were used (Figure 4).

Of the 25 patients, 22 (88%) had a total score of ≤6, considered very close to the results for normal individuals. Only 1 patient reported a score of 9, a value that usually indicates unaesthetical outcomes. The highest possible total score of 13 was not reported by any patient.

Post-operative complications were observed in only 3 patients, a very low rate compared to that reported by other authors [9,10,12,29]; furthermore, no plate fracture was observed [30]. The aforementioned points suggest that this type of surgery requires a detailed knowledge of the relevant anatomical region and a team that is experienced and highly specialized in the surgical treatment of mandibular condylar fractures. These conditions are a prerequisite for reducing the likelihood of post-operative complications, as shown by our results.

In fact, ORIF is a complex surgical procedure for condylar fractures in an area with many anatomical hazards. It may be technically difficult to manipulate and reduce the length of the segments, especially when the condyle is medially displaced. Our evaluation of postoperative radiographs indicated an adequate reduction after fixing any condyle fracture that occurs in the anatomical position within the glenoid fossa. The average degree of patient satisfaction in this study was 8.32 out of 10.

## Conclusion

ORIF could be considered the treatment of choice for patients with neck and sub-condylar mandibular fractures.

## Competing interests

The authors declare that they have no competing interests.

## Authors’ contributions

SA and PP directed the present study. All the authors contributed to the study concept and design. CU and PC helped with article searches, review, and selection. All the authors contributed to the analysis and interpretation of data and drafting of the manuscript. FF, PA, and LG worked as methodological advisors. All authors read and approved the final manuscript.

## Pre-publication history

The pre-publication history for this paper can be accessed here:

http://www.biomedcentral.com/1471-2482/14/68/prepub
